# Supplementary material for: Rare earth metals production using alternative feedstock that eliminates HF
Source: Nat Commun. 2025 May 15;16:4528. doi: 10.1038/s41467-025-59468-w (PMC12081694; doi:10.1038/s41467-025-59468-w)
Supplement: Supplementary file 1 — Supplementary Information [file 41467_2025_59468_MOESM1_ESM.pdf]

Supplementary Information

Rare earth metals production using alternative feedstock that eliminates HF

Anirudha Karati[a], Harshida Parmar[a], Trevor Riedeman[b], Matthew Besser[b], Denis Prodius[a], Ikenna C. Nlebedim[a]

[a]*Division of Critical Materials, Ames National Laboratory, Ames, IA 50011, USA*

[b] *The Materials Preparation Center, Division of Materials Science and Engineering, Ames National Laboratory, Ames, IA 50011, USA*

Preparation of $NdCl_3 \cdot 6H_2O$

The neodymium chloride hydrate is produced from neodymium hydroxide (solid) and hydrochloric acid as follows:

$$Nd(OH)_3(s) + 3HCl\ (aq) \rightarrow NdCl_3(aq) + 3H_2O \qquad (1)$$

However, to obtain $NdCl_3 \cdot 6H_2O$ and to remove any excess HCl, the solution was evaporated until dry. During this drying process, a portion of the neodymium chloride may have transformed to neodymium bis-hydroxy chloride either by Process 1 (equation 2) or Process 2 (Equations 3 and 4) [1]. It is important to note that only a small portion of the $NdCl_3$ may have transformed to $Nd(OH)_2Cl$.

Process 1:

$$NdCl_3 + 2H_2O \rightarrow Nd(OH)_2Cl + 2HCl \qquad (2)$$

Process 2:

$$NdCl_3 \cdot nH_2O \rightarrow NdCl_3 \cdot 2H_2O + (n-2)H_2O \qquad (3)$$

$$NdCl_3 \cdot 2H_2O \rightarrow Nd(OH)_2Cl + 2HCl \qquad (4)$$

Conversion of Nd chloride to fluoride product

However, there is no established reaction for converting neodymium bis-hydroxy chloride to neodymium fluoride upon reaction with sodium fluoride. Liu et al. have recently demonstrated the formation of REF_3 through the conversion of layered rare earth hydroxides intercalated with

various anions [2]. The reaction to produce sodium neodymium fluoride and neodymium fluoride may proceed as follows:

$$Nd(OH)_2Cl + 3NaF \rightarrow NdF_3 + NaCl + 2NaOH \quad (5)$$

$$NdCl_3 \cdot 6H_2O + 4NaF \rightarrow NaNdF_4 \cdot 0.07H_2O + 3NaCl + 5.93H_2O \quad (6)$$

The reactions with neodymium nitrate hexahydrate proceed similarly and are not included for brevity.

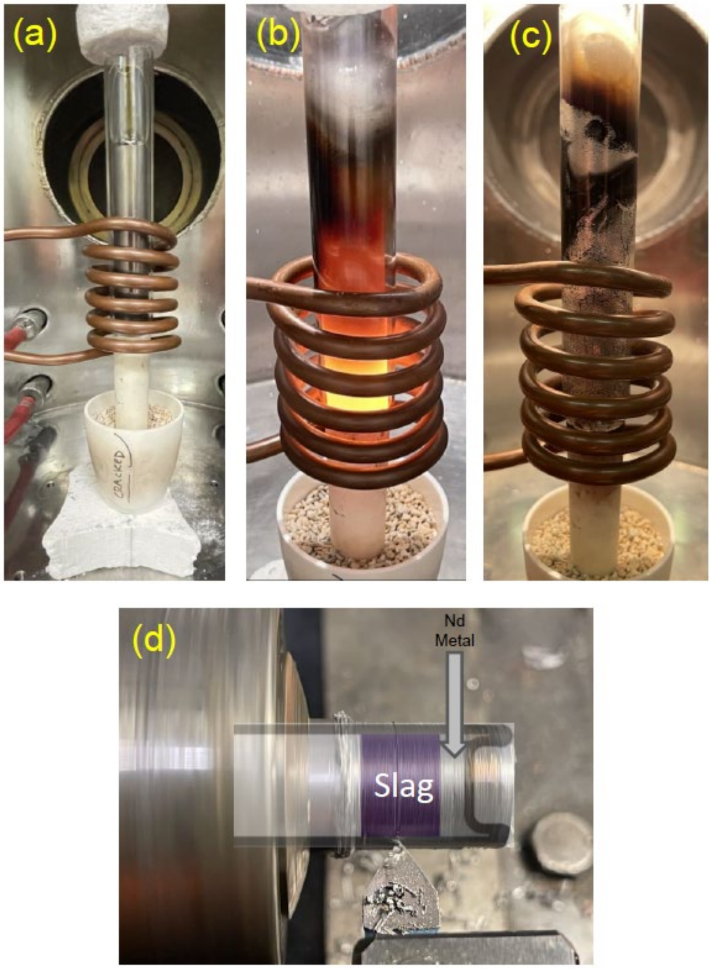

Supplementary Figure 1: Detailed description of the calciothermic processing steps: (a) the charge loaded into a tantalum crucible that is subsequently placed in a quartz tube and suspended into the induction coil; b) the state of the charge when the induction coil reaches 880 °C; c) the state of the charge after the reaction where the calcium gets coated on the inside of the quartz tube; d) the mechanical grinding of the sample to remove the slag.

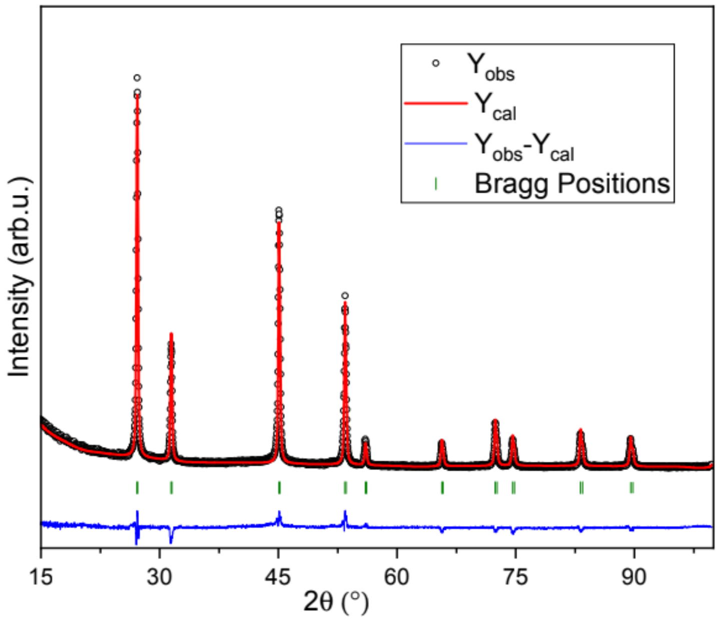

Supplementary Figure 2: Rietveld refined XRD pattern of NNF-A showing single phase α-$NaNdF_4$ [space group: $Fm\overline{3}m$ (no. 225)]. The red line and blue lines depict the calculated pattern and difference plots, the black circle depicts the observed pattern, and the green lines denote the Bragg positions. NNF-A, sample was synthesized using the acetate precursor.

Supplementary Table 1: Rietveld refinement results of XRD patterns of sample NNF-A (sample was synthesized using the acetate precursor).

Phase	Space group	Lattice Parameter (Å)	Atomic coordinates				Reliability factors
			Atom	(x, y, z)	Occ	U_{iso}	
$NaNdF_4$	Fm-3m (No 225)	a=b=c 5.6843	Na	(0, 0, 0)	0.563	0.00156	X^2 = 3.5
			Nd	(0, 0, 0)	0.563	0.01573	R_{Bragg} = 9.8
			F	(¼, ¼, ¼)	1.000	0.01846	R_f=10.1

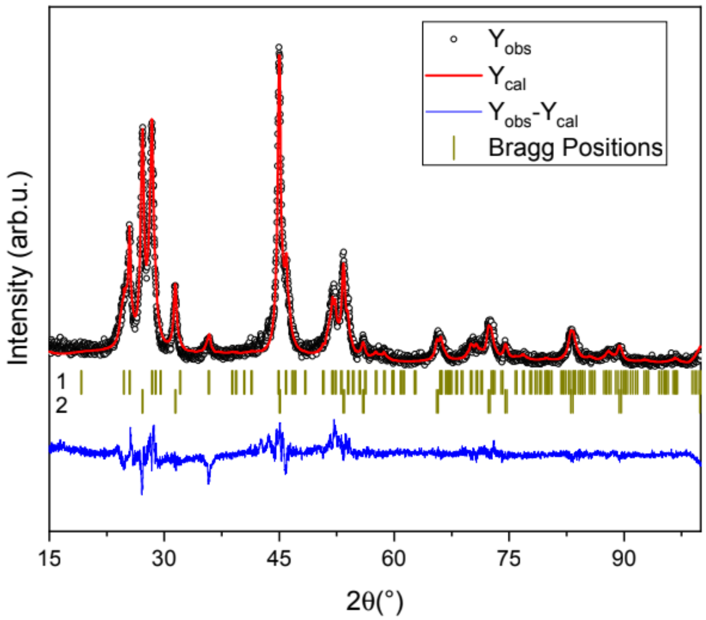

Supplementary Figure 3: Rietveld refined XRD pattern of NNF-C showing two-phase mixture of α-$NaNdF_4$ [space group: $Fm\bar{3}m$ (no. 225)] and NdF_3 [space group: $P6_3/mcm$ (no. 193)]. The red line and blue lines depict the calculated pattern and difference plots, the black circle depicts the observed pattern, and the green lines denote the Bragg positions. Bragg position 1 corresponds to α-$NaNdF_4$ and Bragg position 2 corresponds to NdF_3. NNF-C, sample was synthesized using the chloride precursor.

Supplementary Table 2: Rietveld refinement results of XRD patterns of sample NNF-C (sample was synthesized using the chloride precursor).

Phase	Space group	Lattice Parameter (Å)	Atomic coordinates				Reliability factors
			Atom	(x, y, z)	Occ	U_{iso}	
$NaNdF_4$	Fm-3m (No 225)	a=b=c 5.6855	Na	(0, 0, 0)	0.512	0.00713	χ^2 = 2.7
			Nd	(0, 0, 0)	0.512	0.00422	R_{Bragg}= 2.8
			F	(¼, ¼, ¼)	0.967	0.00667	R_f=2.6
NdF_3	P6322 (No 182)	a=6.6009 c=7.1128	Nd	(1/3, 1/3, 0)	0.500	0.02543	χ^2 = 2.7
			F	(0.380, 0.046, 0.160)	1.000	0.01531	R_{Bragg}= 3.1
			F	(0.333, 0.667, 0.083)	0.340	0.01674	R_f=2.6
			F	(0, 0, 0.250)	0.167	0.06721	

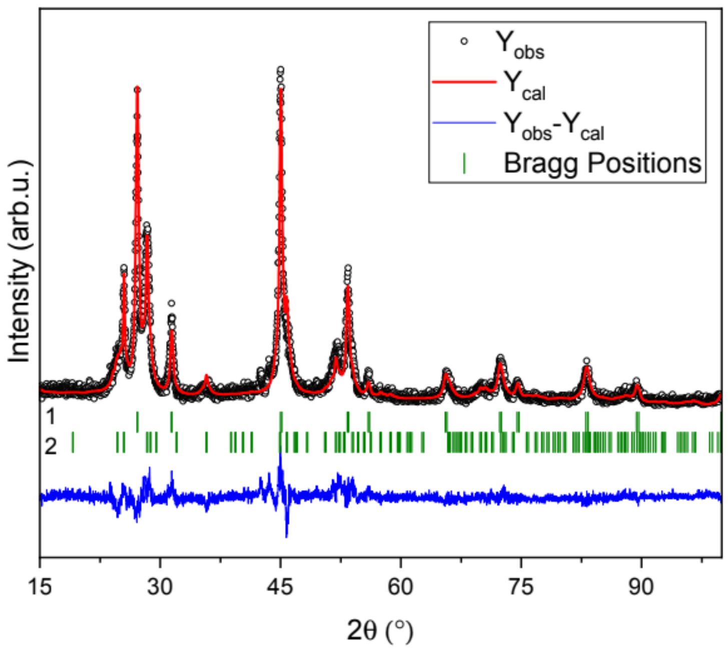

Supplementary Figure 4: Rietveld refined XRD pattern of NNF-N showing two-phase mixture of α-$NaNdF_4$ [space group: $Fm\overline{3}m$ (no. 225)] and NdF_3 [space group: $P6_3/mcm$ (no. 193)]. The red line and blue lines depict the calculated pattern and difference plots, the black circle depicts the observed pattern, and the green lines denote the Bragg positions. Bragg position 1 corresponds to α-$NaNdF_4$ and Bragg position 2 corresponds to NdF_3. NNF-N, sample was synthesized using the nitrate precursor.

Supplementary Table 3: Rietveld refinement results of XRD patterns of sample NNF-N (sample was synthesized using the nitrate precursor).

Phase	Space group	Lattice Parameter (Å)	Atomic coordinates				Reliability factors
			Atom	(x, y, z)	Occ	U_{iso}	
$NaNdF_4$	Fm-3m (No 225)	a=b=c 5.6732	Na	(0, 0, 0)	0.6789	0.00984	χ^2 = 3.4
			Nd	(0, 0, 0)	0.6694	0.00944	R_{Bragg}= 3.5
			F	(¼, ¼, ¼)	0.9421	0.00271	R_f=2.7
NdF_3	P6322 (No 182)	a=6.9920 c=7.2211	Nd	(1/3, 1/3, 0)	0.7479	0.00230	χ^2 = 3.4
			F	(0.380, 0.046, 0.160)	0.8035	0.01703	R_{Bragg}= 6.0
			F	(0.333, 0.667, 0.083)	0.2016	0.07000	R_f=7.7
			F	(0, 0, 0.250)	0.8212	0.00930	

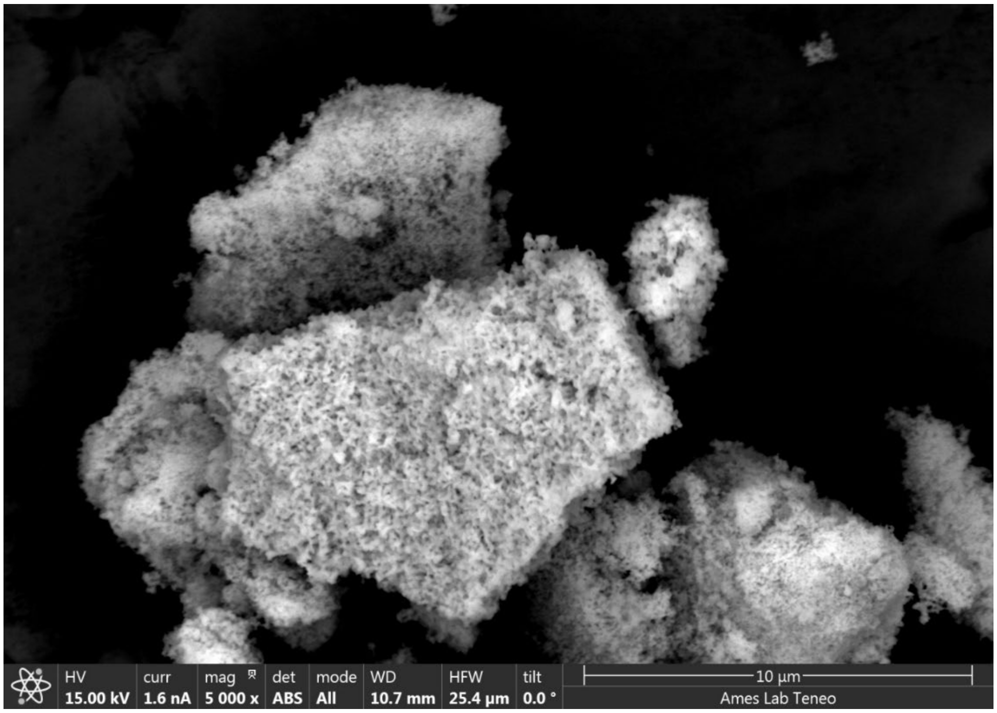

Supplementary Figure 5: Secondary electron micrograph of NNF-A obtained after the reaction of neodymium acetate and sodium fluoride.

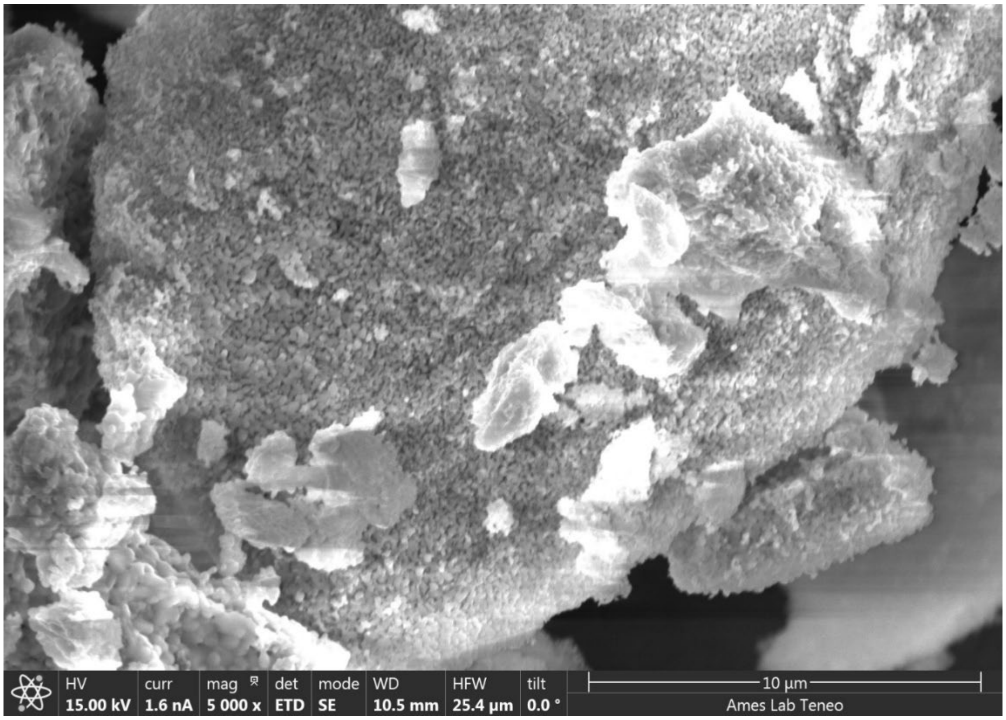

Supplementary Figure 6: Secondary electron micrograph of NNF-C obtained after the reaction of neodymium chloride and sodium fluoride.

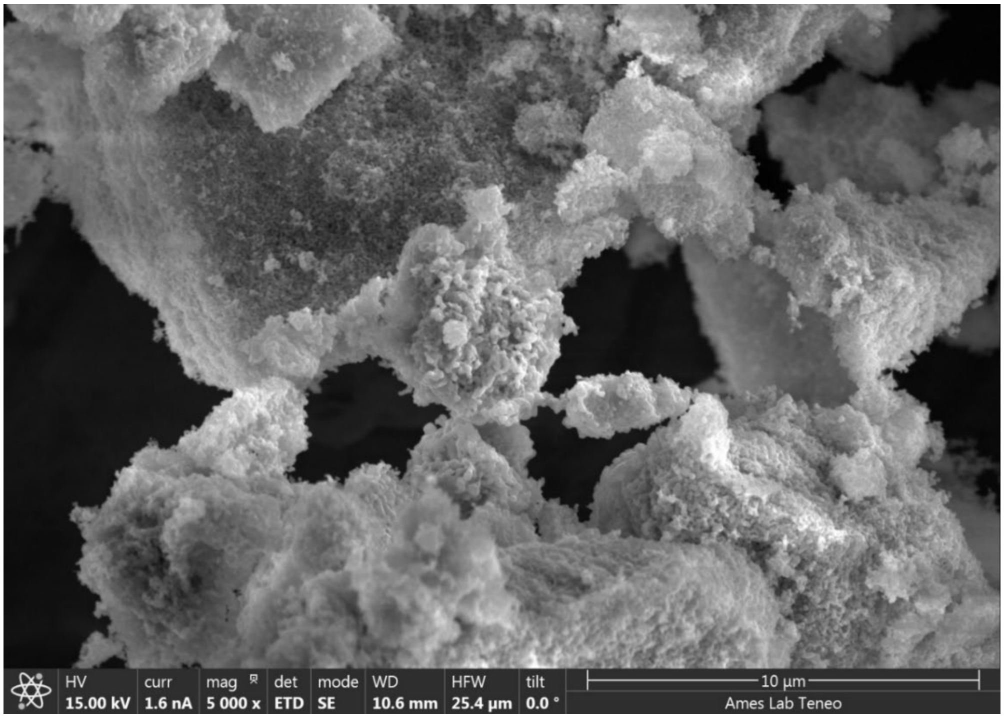

Supplementary Figure 7: Secondary electron micrograph of NNF-N obtained after the reaction of neodymium nitrate and sodium fluoride.

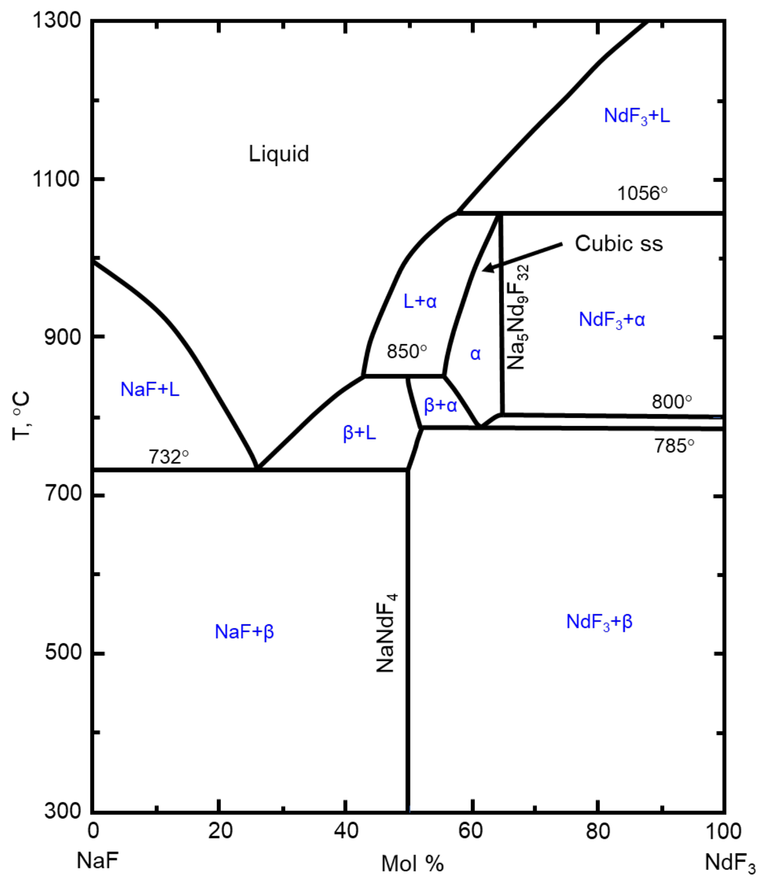

Supplementary Figure 8: Phase diagram of NaF-NdF_3 system. Adapted with permission from THOMA, R. E., INSLEY, H. & HEBERT, G. M. THE SODIUM FLUORIDE-LANTHANIDE TRIFLUORIDE SYSTEMS. INORG CHEM 5, 1222–1229 (1966). Copyright 1966 American Chemical Society [3].

Supplementary References

1. Zehnder, R. A., Clark, D.L., Scott, B.L., Donohoe, R.J., Palmer, P.D., Runde, W.H. & Hobart, D.E. Investigation of the Structural Properties of an Extended Series of Lanthanide Bis-hydroxychlorides $Ln(OH)_2Cl$ (Ln = Nd-Lu, except Pm and Sm). *Inorg Chem* **49**, 4781–4790 (2010).

2. Liu, Z., Golodukhina, S. V., Kameneva, S. V. & Yapryntsev, A. D. Fluorination of Eu-doped layered yttrium hydroxides: the role of anionic composition. *Nanosystems: Physics, Chemistry, Mathematics* **15**, 104–114 (2024).

3. Thoma, R. E., Insley, H. & Hebert, G. M. The Sodium Fluoride-Lanthanide Trifluoride Systems. *Inorg Chem* **5**, 1222–1229 (1966).
